# H_2_ Kinetic Isotope Fractionation Superimposed by Equilibrium Isotope Fractionation During Hydrogenase Activity of *D. vulgaris* Strain Miyazaki

**DOI:** 10.3389/fmicb.2019.01545

**Published:** 2019-07-10

**Authors:** Michaela Löffler, Steffen Kümmel, Carsten Vogt, Hans-Hermann Richnow

**Affiliations:** Department of Isotope Biogeochemistry, Helmholtz Centre for Environmental Research – UFZ, Leipzig, Germany

**Keywords:** hydrogenase, *D. vulgaris* strain Miyazaki, monitoring, GC-IRMS, equilibrium isotope fractionation, kinetic isotope fractionation, hydrogen isotopes

## Abstract

We determined ^2^H stable isotope fractionation at natural abundances associated with hydrogenase activity by whole cells of *Desulfovibrio vulgaris* strain Miyazaki F expressing a NiFe(Se) hydrogenase. Inhibition of sulfate reduction by molybdate inhibited the overall oxidation of hydrogen but still facilitated an equilibrium isotope exchange reaction with water. The theoretical equilibrium isotope exchange δ^2^H-values of the chemical exchange reaction were identical to the hydrogenase reaction, as confirmed using three isotopically different waters with δ^2^H-values of – 62, +461, and + 1533‰. Expected kinetic isotope fractionation of hydrogen oxidation by non-inhibited cells was also superimposed by an equilibrium isotope exchange. The isotope effects were solely catalyzed biotically as hydrogen isotope signatures did not change in control experiments without cells of *D. vulgaris* Miyazaki.

## Introduction

Many microorganisms use hydrogen (H_2_) or protons (H^+^) as electron donors or acceptors, coupled to the oxidation or production of H_2_. The enzyme catalyzing H_2_ oxidation or production is a metalloenzyme termed hydrogenase, for which several differently structured isoenzymes are known (Vignais and Billoud, 2007; Greening et al., 2016). The most abundant and commonly studied type of hydrogenase contains a NiFe(Se)-active center (Vignais and Billoud, 2007). The reaction catalyzed by hydrogenases can be formulated as follows (Eq. 1).

(1) $$H_{2}\Leftrightarrow 2H^{+}+2e^{-}$$

Such a reaction usually leads to a kinetic isotope fractionation, which is defined as the ratio of the rate constants for light and heavy isotopes within the unidirectional reaction of H_2_ to two protons releasing two electrons. The isotope fractionation is a result of the slightly lower activation energy needed to cleave and form bonds of lighter isotopes compared to heavy isotopes in this (bio-) chemical reaction. The kinetic isotope fractionation leads to the predominant reaction of light isotopomers, which implies that the remaining fraction would get heavier during the reaction. For the oxidation of molecular hydrogen at natural abundances, this would result in accumulation of ^2^H (deuterium) in the remaining fraction. However, during the oxidation of H_2_ (Eq. 1), an isotopic exchange with water (Eq. 2) was observed simultaneously to the kinetic isotope effect (Arp and Burris, 1982; Vignais et al., 1997, 2002; Yang et al., 2012).

(2) $$H^{2}H+H_{2}O\Leftrightarrow H_{2}+\;H^{2}HO$$

During the isotope exchange of gaseous hydrogen with water, the heavy isotopes of molecular hydrogen (^2^H) exchange with the light hydrogen isotopes (^1^H) of the water, until an equilibrium isotope value for molecular hydrogen is reached. This is an inverse isotope effect compared to kinetic isotope fractionation, where the H_2_ will become enriched in deuterium over time and thus, the δ^2^H-value will become more positive. If kinetic and equilibrium isotope fractionation take place in parallel, the oxidation and the isotope exchange reaction cannot be separated from each other. In order to understand overall isotope effects during H_2_-consumption, the equilibrium isotope fractionation of the isotopic exchange reaction must be studied separately. Therefore we designed experiments to study both H_2_-oxidation and isotope exchange with cell suspensions of *Desulfovibrio vulgaris* strain Miyazaki F, which expresses one of the best studied NiFe hydrogenases, as well as a NiFe(Se) suited for H_2_ oxidation (Yagi et al., 1976; Deckers et al., 1990; Ogata et al., 2002; Foerster et al., 2003; Fichtner et al., 2006; Pandelia et al., 2010; Nonaka et al., 2013; Riethausen et al., 2013). We hypothesized that the addition of molybdate will inhibit electron flow to sulfate (Peck, 1959; Wolin and Miller, 1980) and thus, only the isotopic exchange reaction would be observable.

The aim of our study was to analyze the equilibrium isotope effect of the isotope exchange reaction and the kinetic isotope effect of the unidirectional oxidation reaction of hydrogen, in order to eventually monitor hydrogenase activity in environmental samples and settings, e.g., during hydrogen underground storage. This method, based on natural abundant stable hydrogen isotopes of gaseous samples, would allow *in situ* assessment of hydrogenase activity without further treatment of samples.

## Materials and Methods

### Chemicals

All chemicals until otherwise stated were purchased from Merck Chemicals GmbH (Darmstadt, Germany). Deuterium-enriched waters were prepared by mixing 1 l sterilized tap water water (Merck Millipore, Germany) with either 250 μl or 100 μl ^2^H_2_O (99.9%; Merck Chemicals, Germany).

### Culture and Cultivation Conditions

*Desulfovibrio vulgaris* strain Miyazaki F (DSM 19637) was obtained from the DSMZ (Deutsche Sammlung von Mikroorganismen und Zellkulturen, Braunschweig, Germany). The strain was grown in a mineral medium for sulfate-reducers, which consisted of NH_4_Cl (0.3 g/l), KH_2_PO_4_ (0.4 g/l), CaCl_2_ (0.075 g/l), Na_2_SO_4_ (2 g/l), MgSO_4_^∗^ 7 H_2_O (1 g/l), 1 ml trace element solution SL-10, 0.1 ml selenite-tungstate solution, 4 ml 50 % Na-DL-lactate, 2 ml vitamin solution, 10 ml 1 M NaHCO_3_ solution, 2 ml 1 M Na-acetate solution. 1 M L-cysteine solution was used for reduction. 0.1 mg/l resazurin was used as redox indicator. The selenite-tungstate solution contained per 100 ml: 0.5 g/l NaOH, 3 mg/l Na_2_SeO_3_^∗^ 5 H_2_O and 4 mg/l Na_2_WO_4_^∗^ 2 H_2_O. The vitamin solution contained per 100 ml: 1 mg biotin, 1 mg folic acid, 25 mg pyridoxine-HCl, 25 mg thiamine-HCl ^∗^ 2 H_2_O, 5 mg riboflavin, 25 mg nicotinic acid, 25 mg D-Ca-pantothenate, 0.5 mg vitamin B_12_, 25 mg p-aminobenzoic acid and 25 mg lipoic acid. All components, except the last four, were mixed in sterilized tap water (Merck Millipore, Germany) and purged with 75% N_2_ and 25% CO_2_ until they became virtually oxygen-free and were autoclaved subsequently. The remaining components were added within an anaerobic glovebox (Toepffer Lab Systems, Germany).

Experiments with cell suspensions were performed with cells pre-grown on 28 mM lactate and 22 mM sulfate at 30°C and 120 rpm on a Multitron incubation shaker (Infors, Germany). At optical densities above 0.2 absorbance with no further increase in cell density, 50 ml each were transferred into 120 ml serum bottles in the glovebox, which was filled with a mixture of N_2_ (97–98%) and H_2_ (2–3%) and crimped close with PTFE-coated chlorobutyl-isoprene septa (Thermo Fisher Scientific, Germany) before the start of the experiment.

Three sets of isotope fractionation experiments were designed with these cells: one set in which cells were inhibited by molybdate, one in which the headspace had been purged to reduce sulfide load, and one without further treatment. For the inhibited setups, six of the bottles each were treated with 20 mM molybdate. Therefore, 2 ml of a 0.5 M sodium molybdate solution was added to each bottle, yielding yellow-orange molybdo-sulfide-complexes (Wolin and Miller, 1980; Biswas et al., 2009). Both the purged and untreated setups consisted of five bottles of cell suspension each. The headspace in the purged setups was exchanged with N_2_ to eliminate H_2_S-burden in the headspace and reduce potential inhibition by sulfides.

In all samples 12 ml H_2_ were hereafter exchanged with the existing headspace with a syringe and an empty needle while bottles were tilted to remove headspace only. Bottles were kept at 30°C and 120 rpm (Multitron incubation shaker, Infors, Germany). For abiotic controls cell suspensions were inactivated by incubation at 80°C in a water bath for 20 min and subsequent addition of 0.3 ml 10 M NaOH, yielding pH 12.

All experimental setups were performed in two replicates using water at natural abundance (δ^2^H-H_2_O = -62‰) and water enriched in ^2^H by adding 100 μl ^2^H_2_O resulting in an isotope composition of δ^2^H-H_2_O = +461‰ One experiment with molybdate for inhibition of sulfate reduction was conducted with enriched water (250 μl ^2^H_2_O) to yield a final isotope composition of δ^2^H-H_2_O = + 1533‰. Bottles were sacrificed at different time points in a water bath at 80°C for 20 min and stored upside down after the addition of 10 M NaOH before hydrogen isotopes of H_2_ in the headspace were analyzed. The setup with enriched water (δ^2^H-H_2_O = + 1533‰) was sacrificed in different periods of time than the other experiments, in order to gain insight into the initial reaction. NaOH was added to terminate microbial activity and to remove CO_2_ from the headspace by precipitation of sodium bicarbonate. CO_2_-removal was necessary for subsequent isotope analysis, as the column used for gas chromatography (GC) would retain it. 100 to 300 μl sample volume were transferred via syringe into 1 ml 3% (w/v) Zn-acetate and stored at -20°C for downstream processing. Sulfides in the headspace of the bottles were eliminated due to precipitation as ZnS by the addition of 3 ml 3 (w/v) % Zn-acetate.

### Analytical Methods

#### Sulfide Measurements

Sulfides were measured as previously described (Cline, 1969; Kleinsteuber et al., 2008) against a blank control. Previously frozen samples were thawed and mixed with 4 ml water and 0.4 ml of Cline’s solution. The mixtures were stored in the dark for 20 min before photometric measurement on an UV-1800 (Shimadzu, Germany) at 670 nm. Due to the high concentrations of sulfides, samples were diluted before measurement. Optical densities of cell suspensions were determined on an UV-1800 (Shimadzu, Germany) at 600 nm. 1 ml culture solution was transferred via syringe into a cuvette filled with a few mg of Na-dithionite, closed with parafilm and shaken until dissolved and then measured using water as control.

#### Isotope Measurement

Hydrogen isotope measurements were performed on a GC-isotope ratio mass spectrometer (IRMS) system (Supplementary Figure 1) (7890A, Agilent Technologies, Germany; GC-IsoLink II Thermo Fisher Scientific, Germany; Conflo IV Thermo Fisher Scientific, Germany; Finnigan MAT 253, Thermo Fisher Scientific, Germany) equipped with a J&W CP-Molsieve 5Å GC Column (50 m, 0.32 mm, 30 μm, Agilent Technologies, Germany). An empty ceramic tube was kept isothermal at 500°C inside the GC-IsoLink and the GC column was kept at 40°C during measurements. An additional cold trap operated with liquid nitrogen was installed after the GC to remove water to keep the water background within the ion source of the IRMS low. After 2 h of runtime, the column was heated to 250°C and held for at least 10 min remove residual water vapor and residual CO_2_ from the GC column.

0.2 ml headspace injections were made manually at split-ratio of 1:25 with a gas-tight syringe in an interval of roughly eight minutes needed for elution of H_2_ and permanent gases. The analytical system was evaluated for reproducibility and isotope artifacts. It was found to be reproducible and deliver true values within the uncertainty of 0.7 ± 0.4‰.

Both the normal and the δ^2^H-enriched waters were measured with an elemental analysis-chromium/high temperature conversion (EA-Cr/HTC)-IRMS system (HEKAtech, Germany) coupled via the Conflo IV (Thermo Fisher Scientific, Germany) to the same IRMS instrument (Gehre et al., 2017). All results are reported in the delta notation (Eq. 3) and according to the guidelines for stable isotope measurements (Coplen, 2011).

(3) $$\delta [‰]=\left(\frac{R_{\mathrm{sample}}}{R_{\mathrm{standard}}}-1\right)*1000$$

The ratio of heavy to light isotopes (R_*sample*_) in a compound is reported in δ-notation; for hydrogen, samples will be compared to Vienna Standard Mean Ocean Water (VSMOW) as standard with an isotope ratio of 155.76 ± 0.1 ppm (R_*standard*_). A laboratory standard, made of 10% H_2_ in N_2_, with a δ^2^H-value of -205‰ was used for reference. The auto-protonation factor (H_3_^+^ factor) was determined daily and remained stable at 8.24 ± 0.05. Theoretical equilibrium isotope values, mainly dependent on temperature and the isotope signature of water, were calculated according to a formula previously described (Horibe and Craig, 1995).

#### Assessment of H_2_ Concentrations

The concentration of H_2_ was measured with the GC-IRMS. For concentration measurements a defined volume of 10% H_2_ was prepared using 120 ml serum bottles which were purged with N_2_ before. Twelve ml H_2_ were exchanged with the gaseous phase by a syringe and an empty needle while the bottle was held upside down. This sample was prepared daily and used for external calibration of the concentration measurement. Therefore the sample was injected three times at the start of each run, and all following samples were normalized to the average area under the curve of all controls in this measurement period. Intensities of external calibration were stable over 20 days, leading to overall variations in H_2_-concentration of ± 1.5%. Initial concentrations of H_2_ between setups and replicates varied about 5% of the response, probably dependent on handling speed and temperature in the laboratory during exchange of H_2_, as well as gas-tightness of the syringe used for injections.

## Results

### Measurement of Hydrogen Stable Isotopes

The isotope signatures of the different waters were determined to be δ^2^H-H_2_O = -62 ± 2‰, +461 ± 1‰, and +1533 ± 2‰, respectively. For the sterilized tap water in Leipzig (δ^2^H-H_2_O = -62‰), an equilibrium δ^2^H-value for H_2_ of – 744‰ was calculated. The enriched water setups (δ^2^H-H_2_O = + 461‰ and δ^2^H-H_2_O = + 1533‰) were calculated to equilibrate with a theoretical value for H_2_ of δ^2^H = -606‰ and δ^2^H = -317‰, respectively.

**FIGURE 1:**
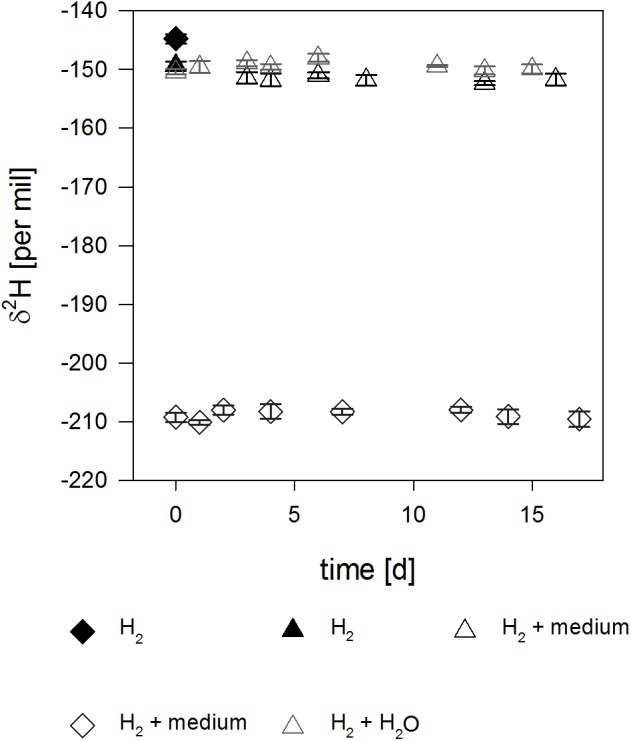
Isotope signal over time in the abiotic controls. Filled symbols correspond to pure H_2_ used for the experiment and empty symbols correspond to H_2_ in the headspace of serum bottles filled with either water (gray) or medium (black). Please note the depleted δ^2^H-values of H_2_ (empty diamonds) for the medium prepared in the anaerobe glovebox due to mixing of two isotopically different H_2_ sources (see text).

Control experiments with H_2_ in the headspace and sterilized tap water or culture medium showed no significant change in the isotopic signature over the experimental timeframe of 18 days (Figure 1), with stable δ^2^H-values of δ^2^H = -141.2‰ and δ^2^H = -142.2‰. These were identical to the isotope value of the pure H_2_ used (δ^2^H = -139‰), taking usual uncertainties into account. The δ^2^H signature of the hydrogen gas used in the anaerobic glove box was substantially lighter (approx. δ^2^H = -645‰) than the signature of the H_2_ gas used in the whole cell experiments, leading to a shift of Δ^2^H = 65‰ (Figure 1) when mixed.

### H_2_-Oxidation by Non-inhibited Cells

#### Experiments With Reduced Sulfide Concentration

To reduce concentrations of sulfide which may affect the metabolism of *D. vulgaris*, the headspace of culture bottles were purged with N_2_. The sulfide concentrations after purging were typically roughly 6 mM.

**FIGURE 2:**
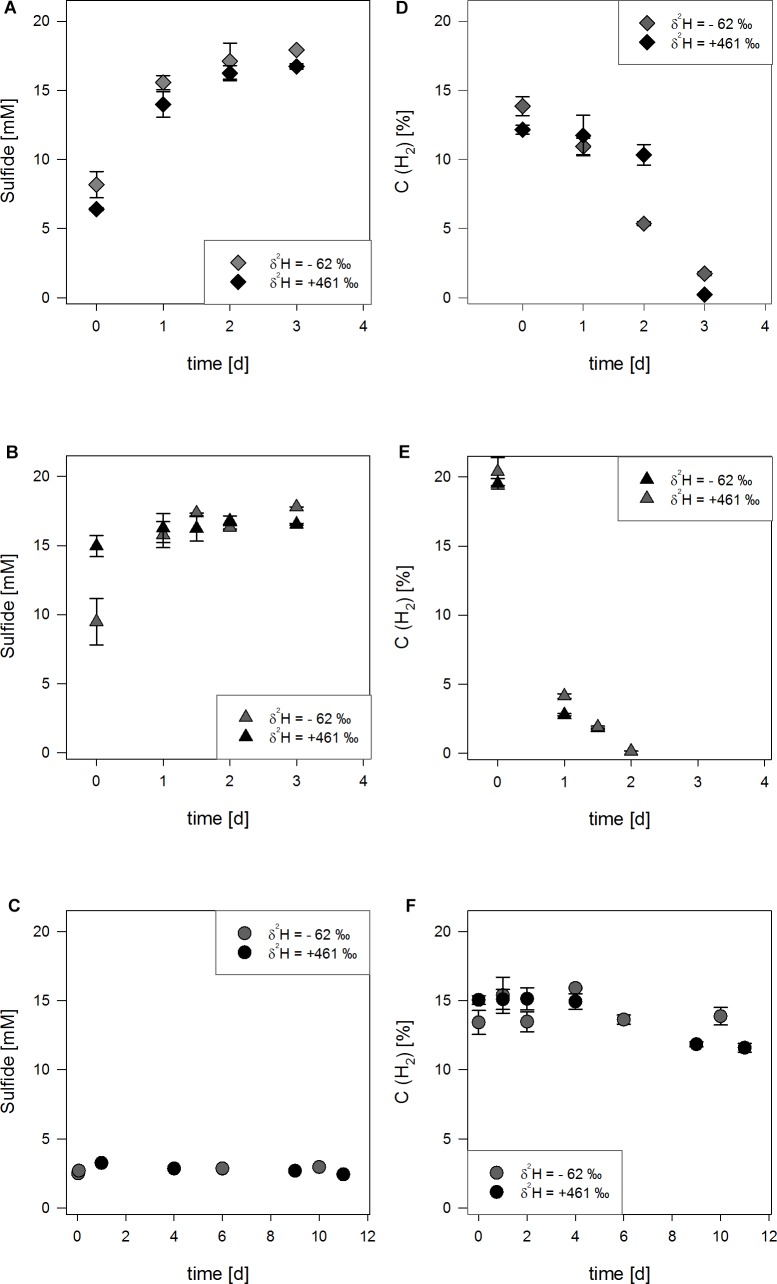
Concentration of H_2_ (empty symbols) and sulfides (filled symbols) for three experimental setups **(A,D)**: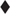 setups with reduced sulfide burden, **(B,E)**: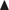 setups without further treatment, **(C,F)**: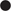 inhibition by molybdate) in either water with δ^2^H_2_O = -62‰ (gray) or with δ^2^H_2_O = +461‰ (black). The uncertainty of 2σ is shown.

**FIGURE 3:**
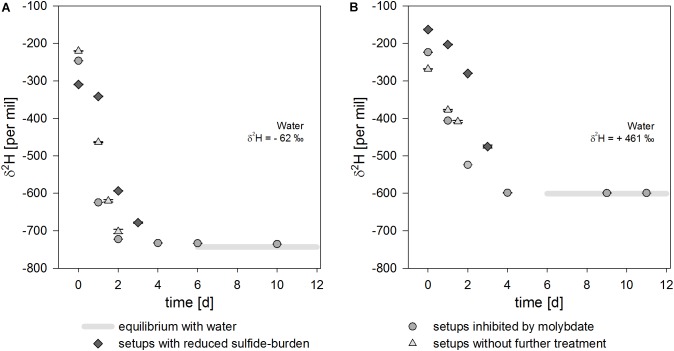
Isotope signal over time for three experimental setups in either water with δ^2^ H_2_O = –62‰ **(A)** or with δ^2^ H_2_O = +461‰ **(B)**. The isotope equilibrium is reached after about 6 days and indicated by light gray bars. The uncertainty of 2σ is shown and often smaller than the symbol.

13.9 ± 0.7% and 12.2 ± 0.3% H_2_ were oxidized within 4 days (Table 1 and Figure 2D). Sulfide concentrations increased from 6.0 ± 2.2 mM and 6.4 ± 0.1 mM to 17.9 mM and 16.7 ± 0.2 mM, respectively, in 3 days (Figure 2A). Hydrogen isotope values changed slowly toward depletion in the beginning from δ^2^H = -309.0 ± 0.7‰ and δ^2^H = -163.2 ± 0.9‰, before the reaction gained speed (Table 2 and Figure 3), yielding δ^2^H = -678.6 ± 1.5‰ and δ^2^H = -475.3 ± 4.1‰ after 3 days.

**Table 1:** Concentration of H_2_ in all experimental setups: molybdate-inhibited, headspace purged with N_2_ to reduce sulfides and without additional treatment.

time	δ^2^H = -62‰						δ^2^H = +461‰						δ^2^H = +1533‰	
			
	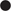 Molybdate		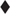 Purged		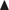 Untreated		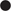 Molybdate		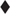 Purged		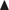 Untreated		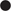 Molybdate	
							
	C [%]	Stdev [%]	C [%]	Stdev [%]	C [%]	Stdev [%]	C [%]	Stdev [%]	C [%]	Stdev [%]	C [%]	Stdev [%]	C [%]	Stdev [%]
1 min	13.4	0.9	13.9	0.7	20.4	1.0	15.0	0.3	12.2	0.3	19.5	0.4		
29 min													18.2	1.0
55 min													19.1	0.3
88 min													20.0	0.2
3 h 40 min													14.5	0.2
1 day	15.4	1.3	10.9	0.6	4.1	0.2	15.1	0.7	11.7	1.5	2.8	0.1		
1.5 day					1.9	0.1					1.8	0.1		
2 days	13.5	0.7	5.4	0.1	n.d.	n.d.	15.1	0.8	10.3	0.7	n.d.	n.d.		
3 days			1.7	0.1	n.d.	n.d.			0.2	0.0	n.d.	n.d.	13.5	1.0
4 days	15.9	0.1	n.d.	n.d.			14.9	0.6	n.d.	n.d.				
6 days	13.6	0.3											13.4	2.1

9 days							11.8	0.2						

10 days	13.9	0.6											11.6	0.1
11 days							11.6	0.3										
Average	14.3	1.3					13.9	1.7					15.8	3.3


**Table 2:** δ^2^H-values of H_2_ in all experimental setups: molybdate-inhibited, headspace purged with N_2_ to reduce sulfides and without additional treatment.

time	δ^2^H = -62‰						δ^2^H = +461‰						δ^2^H = +1532‰	
			
	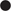 Molybdate		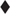 Purged		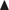 Untreated		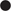 Molybdate		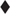 Purged		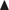 Untreated		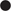 Molybdate	
							
	δ^2^H [‰]	Stdev [‰]	δ^2^H [‰]	Stdev [‰]	δ^2^H [‰]	Stdev [‰]	δ^2^H [‰]	Stdev [‰]	δ^2^H [‰]	Stdev [‰]	δ^2^H [‰]	Stdev [‰]	δ^2^H [‰]	Stdev [‰]
1 min	-246.5	0.7	-309.0	0.7	-220.4	0.7	-223.9	0.5	-163.2	0.9	-268.4	0.5		
29 min													-162.4	1.7
55 min													-154.1	0.6
88 min													-154.7	1.0
3 h 40 min													-177.4	1.4
1 day	-624.7	0.3	-341.0	1.0	-463.8	1.2	-405.7	0.3	-203.3	1.0	-377.6	2.3		
1.5 day					-620.2	3.2					-408.1	2.8		
2 days	-722.8	0.4	-593.6	0.8	-702.1	5.7	-524.1	0.3	-280.2	0.4	n.d.	n.d.		
3 days			-678.6	1.5	n.d.	n.d.			-475.3	4.1	n.d.	n.d.	-279.8	0.3
4 days	-733.2	0.3	n.d.	n.d.	n.d.	n.d.	-598.9	0.4	n.d.	n.d.	n.d.	n.d.		
6 days	-733.8	0.3											-328.0	0.7
9 days							-599.6	0.4						
10 days	-735.8	0.3											-327.3	0.3
11 days							-599.4	0.1						


#### Experiments With High Sulfide Concentration

In the experiments without further treatment to reduce the amount of sulfides present, 20.4 ± 1.0% H_2_ and 19.5 ± 0.4% H_2_, respectively were consumed within 2 days in normal water (Table 1 and Figure 2E). 17.8 mM and 16.5 mM sulfides were produced starting from 9.48 ± 1.7 mM and 11.2 ± 0.6 mM until day three (Figure 2B). The δ^2^H of hydrogen in the bottles’ headspace was rapidly decreasing from δ^2^H = -220.4 ± 0.7‰ and δ^2^H = -268.4 ± 0.5‰ to δ^2^H = -702.1 ± 5.7‰ and δ^2^H = -408.1 ± 2.8‰ after 1.5 to 2 days (Table 2 and Figure 3). Concentrations at the last measureable time-points were 1.9 ± 0.1% and 1.8 ± 0.1% H_2_.

#### H_2_ Isotope Exchange by Cells Inhibited With Molybdate

Molybdate was used to inhibit the electron flow to the electron acceptor sulfate. In molydate-amended cultures, the H_2_ concentrations were almost constant with 14.3 ± 1.3%, 13.9 ± 1.7% and 15.8 ± 3.3% H_2_. Thus, no consumption of H_2_ in the molybdate setups was observed (Table 1 and Figure 2F). Furthermore, the sulfide concentrations did not increase and were stable between 2.5 to 3.5 mM (Figure 2C), indicating that sulfate reduction to sulfide was completely inhibited. In all experimental setups inhibited by molybdate, hydrogen isotopes in the headspace were depleting in deuterium starting from δ^2^H = -246.5 ± 0.7‰, δ^2^H = -223.9 ± 0.5‰, and δ^2^H = -162.4 ± 1.7‰ (Table 2) and stabilized at δ^2^H = -735.8 ± 0.4‰, δ^2^H = -599.4 ± 0.1‰, and δ^2^H = -327.3 ± 0.3‰ for the differently enriched waters after 6 days (Figure 3).

## Discussion

No changes in the isotope signature of H_2_ for both culture medium and water in the abiotic controls could be observed, even though H_2_-concentrations decreased with continuous sampling of the same bottles (Supplementary Figure 2). Most studies on hydrogen isotope exchange with water used platinum or palladium as a catalyst and subsequent equilibration times of a few hours were reported at, e.g., 20°C (Crist and Dalin, 1934; Farkas and Farkas, 1934; Horiuti and Polanyi, 1934; Farkas, 1936; Suess, 1949; Rolston et al., 1976). It is therefore reasonable to assume that isotope exchange without a catalyst is too slow to be assessable in the experimental timeframe used in this study. Subsequently, it is unlikely that the isotope signal in environmental samples could be significantly affected by minerals, as catalysts are needed for accelerating the exchange reaction. But sampling itself might lead to bias, as it has been shown that use of steel can lead to the generation of molecular H_2_ from water, with which it is equilibrated (Chapelle et al., 1997).

Addition of gaseous hydrogen to water or culture medium leads to an immediate shift in the isotope signature from δ^2^H = -139.0 ± 0.8‰ to δ^2^H = -141.2 ± 0.3‰ for water and δ^2^H = -142.2 ± 0.7‰ for culture medium. This effect is probably due to a relatively higher solubility of ^2^H in water, which leads to an isotope fractionation (Muccitelli and Wen, 1978). The observed shift in isotopic signature lies within usual standard deviations for hydrogen isotope measurements, and no further change in the isotope signal was observed after the initial shift (Figure 1). Albeit negligible compared to biocatalysis, isotope exchange takes place in abiotic controls within 18 days at 30°C in the absence of a catalyst.

The addition of a cell suspension leads to changes in the isotope signature due to hydrogenase activity. An inverse isotope effect was observed in all three experimental setups. This is consistent with previously reported δ^2^H -values for H_2_-production from water for *Shewanella oneidensis* MR-1, which can express a NiFe- or a FeFe-hydrogenase (Kreuzer et al., 2014). Upon inhibition of dissimilatory sulfate reduction by molybdate, observable isotope effects should be limited to isotope exchange, since H_2_ was not consumed. Hydrogenases were previously described to facilitate isotope exchange of ^2^H_2_ and H_2_O or H_2_ and ^2^H_2_O (Hoberman and Rittenberg, 1943; Jouanneau et al., 1980; Arp and Burris, 1982; Vignais et al., 2000, 2002). We therefore assume that the hydrogenase in our experiments is solely responsible for the isotope exchange.

**FIGURE 4:**
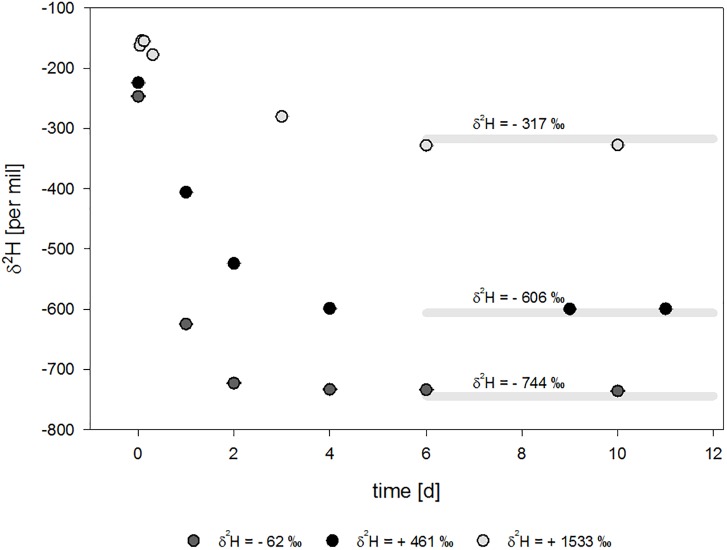
Isotope signature over time for molybdate-inhibited setups with water of different isotope composition. Theoretical equilibrium values ± approx. 5‰ standard deviation are indicated by light gray lines.

After four to 6 days the isotope exchange reaction approximately reached equilibrium with differences of Δ^2^H = 9‰ and Δ^2^H = 6‰ and Δ^2^H = 10‰ compared to the theoretical values (Figure 4) in cultures inhibited by molybdate addition. Deviations from the theoretical values could be due to the higher measurement error and standard deviations of the water measurements used for calculation, as well as the high sensitivity of the equilibrium equation toward fluctuations in temperature.

It has been shown for the NiFe-hydrogenase from *D. vulgaris* Miyazaki that hydrogen oxidation is a two-step process, during which an enzyme-hydride-state forms (Lubitz et al., 2014). First, H_2_ diffuses into the active center. Then, it is heterolytically cleaved, forming a proton and an enzyme-hydride-complex. Afterward, electrons and protons are shuffled out of the protein structure (Lubitz et al., 2014). A study using *D. vulgaris* Hildenborough suggested that H_2_ can be caged by the protein structure surrounding the active site when selenocysteine replaces cysteine in the active center of the protein structure (Gutiérrez-Sanz et al., 2013). In this case, substrate and products can accumulate within the protein structure of the NiFeSe-hydrogenase near the catalytic center and the two transfers of protons proceed faster than in NiFe-hydrogenases. The authors suggested that the accumulation and subsequent availability of substrate would correspond to a fast isotope exchange reaction (Gutiérrez-Sanz et al., 2013). This hypothesis connects protein structure and isotope effects. *D. vulgaris* Miyazaki expresses a structurally similar NiFeSe-hydrogenase best suited for hydrogen oxidation (Nonaka et al., 2013; Riethausen et al., 2013), and the corresponding fast isotope exchange reaction was measured in this study, where the isotope equilibrium was reached within 6 days. It might be possible to compare the equilibrium isotope effect of structurally different types of hydrogenases, such as NiFe- and FeFe-hydrogenases, in order to characterize the isotope exchange rate in future studies in more detail. For this, a different inhibition of electron flow might be needed, as molybdo-sulfide-complexes might not work for all microorganisms, as it specifically inhibits sulfate reduction. Even though it has been shown to also inhibit H_2_ production from glucose (Wolin and Miller, 1980), it has not been further or sufficiently studied. Information on structurally different hydrogenases is crucial for using stable hydrogen isotopes as a monitoring tool to track *in situ* hydrogenase activity, e.g., during storage of hydrogen in underground reservoirs.

Even though H_2_ was consumed in the experiment with purged headspaces, only minimal changes in the δ^2^H-values were observed in the beginning. During the substrate consumption, the hydrogen bond is cleaved, which is expected to result in a normal kinetic isotope effect. However, in our experiments, the isotope values approximate isotope equilibrium values with increasing time. We therefore suggest that the kinetic isotope effect of the hydrogen bond cleavage is superimposed by the equilibrium isotope exchange reaction. Only in the beginning of hydrogen oxidation, an effect of the kinetic isotope effect can be observed. Here, the equilibrium isotope effect has not yet completely superimposed the isotope signature, which results in seemingly stable isotope values.

The observed kinetic isotope fractionation effects could be due to shuffling of protons into the cell. Hydrogenase and cytochrome complexes are able to translocate protons (Ide et al., 1999; Dolla et al., 2000; Chang et al., 2004). For example, the reduction of sulfate needs two additional protons (Eq. 4).

(4) $$4H_{2}+SO_{4}^{2-}+2H^{+}\to H_{2}S+4H_{2}O$$

During this process a slight isotope fractionation is expected, due to different diffusivity according to their molecular mass and tunneling effects in the hydrogenase structure (Cukier, 2004). Then, more ^2^H^+^ than ^1^H^+^ would be released from the hydrogenase, which would equivalent the expected kinetic isotope fractionation. This effect would counteract an equilibrium isotope exchange reaction. Not only the rate of equilibrium isotope exchange, but also the kinetic isotope fractionation could be affected by the protein structure. Therefore, further studies on both kinetic isotope effects and equilibrium isotope effects and their superimposition using structurally different hydrogenases are needed in order to use this concept as a monitoring or diagnostic tool.

Interestingly, cultures containing the highest sulfide concentrations tested in this study (10 mM) showed consumption of H_2_, but lacked the “isotopic lag phase” of the purged experiments with 6 mM starting concentration (Figure 3). This might be an indication that the amount of sulfides could affect the electron and proton flow, as H_2_ is still consumed but the normal kinetic isotope fractionation of the H-H bond cleavage is immediately superimposed by the equilibrium isotope effect of the exchange reaction. During growth on lactate and sulfate, up to 52% of electrons flow into the production of H_2_ and the remaining 48 % of electrons are coupled to sulfate reduction in *D. vulgaris*, yielding the potential to reduce approx. 8.9 mM sulfate from lactate and H_2_ (Noguera et al., 1998) or 14 mM sulfate from lactate alone before the start of the hydrogen oxidation experiments. And with concentrations of 9.48 up to 11.2 mM sulfide in the setups without further treatment to reduce sulfides at the start of the experiment, inhibition is a consequential hypothesis.

## Conclusion

The hydrogenase of *Desulfovibrio vulgaris* Miyazaki facilitates an equilibrium isotope exchange when consumption of H_2_ is inhibited. Resulting δ^2^H-values of H_2_ corresponds to theoretical thermodynamic isotope equilibria. During H_2_, the kinetic isotope fractionation, which should be observed due to bond-cleavage, is superimposed by the equilibrium isotope exchange. These results might differ for other microorganisms and structurally different hydrogenases. Equilibrium isotope exchange in the experiments with starting concentration of about 10 mM sulfides also indicates a possibility that sulfides could inhibit electron flow. This research is fundamental in nature and aims to build a better understanding of the isotope effects and processes associated with hydrogenases. The results of this study serve as a basis for future research on a simple monitoring tool for environmental, gaseous samples based on stable isotopes of hydrogen.

## Data Availability

All datasets generated for this study are included in the manuscript and/or the Supplementary Files.

## Author Contributions

ML planned and performed the experiments and data analyses and wrote the manuscript. SK helped to establish a GC-IRMS method for H_2_. CV and H-HR supervised the research and edited the manuscript.

## Conflict of Interest Statement

The authors declare that the research was conducted in the absence of any commercial or financial relationships that could be construed as a potential conflict of interest.
