# Supplementary material for: H2 Kinetic Isotope Fractionation Superimposed by Equilibrium Isotope Fractionation During Hydrogenase Activity of D. vulgaris Strain Miyazaki
Source: Front Microbiol. 2019 Jul 10;10:1545. doi: 10.3389/fmicb.2019.01545 (PMC6636216; doi:10.3389/fmicb.2019.01545)
Supplement: Supplementary file 1 [file Data_Sheet_1.docx]

Supplementary Material

H_2_ kinetic isotope fractionation superimposed by equilibrium isotope fractionation during hydrogenase activity of *D. vulgaris* strain Miyazaki

Michaela Löffler^1^, Steffen Kümmel^1^, Carsten Vogt^1, *^, Hans-Hermann Richnow^1^

^1^Department Isotope Biogeochemistry, Helmholtz Centre for Environmental Research – UFZ, Leipzig, Germany

**
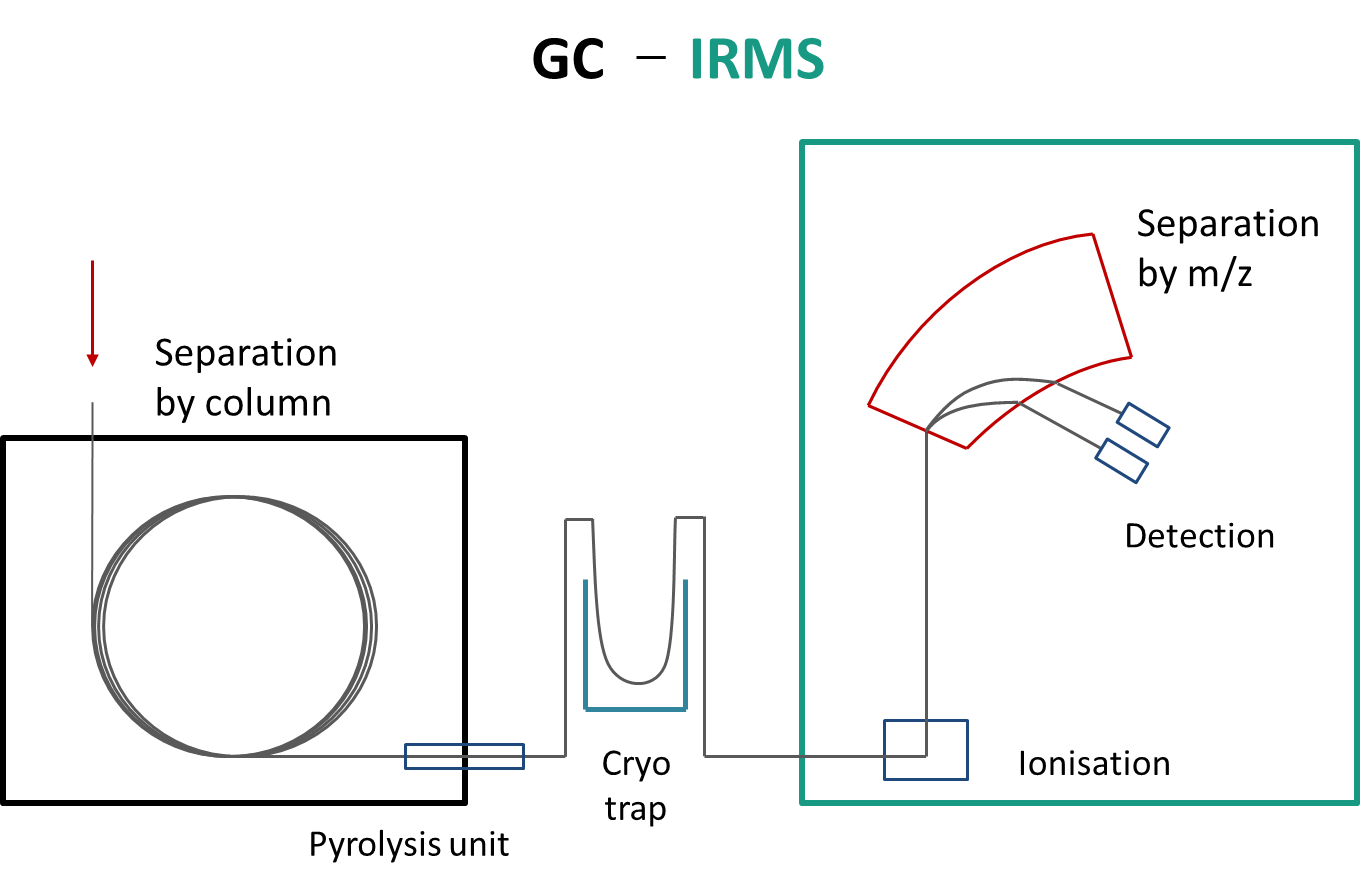
Supplementary Figure 1:** Scheme of the GC-IRMS system

**Supplementary Figure 2:** Concentration of H_2_ of the abiotic controls of both medium and water. Continuous sampling results in decreasing concentrations from 18 % to 10 % H_2_.
